# Enhancing narrative clinical guidance with computer-readable artifacts: Authoring FHIR implementation guides based on WHO recommendations

**DOI:** 10.1016/j.jbi.2021.103891

**Published:** 2021-10

**Authors:** Jennifer Shivers, Joseph Amlung, Natschja Ratanaprayul, Bryn Rhodes, Paul Biondich

**Affiliations:** a Global Health Informatics, Regenstrief Institute, Indianapolis, IN, United States; b Public Digital Health Technology, Department of Digital Health and Innovation, World Health Organization, Geneva, Switzerland; c Alphora, Orem, UT, United States

**Keywords:** Fast Healthcare Interoperability Resources (FHIR), Global Health, Medical Informatics, Practice Guideline, Terminology

## Abstract

**Introduction:**

Narrative clinical guidelines often contain assumptions, knowledge gaps, and ambiguities that make translation into an electronic computable format difficult. This can lead to divergence in electronic implementations, reducing the usefulness of collected data outside of that implementation setting. This work set out to evolve guidelines-based data dictionaries by mapping to HL7 Fast Health Interoperability Resources (FHIR) and semantic terminology, thus progressing toward machine-readable guidelines that define the minimum data set required to support family planning and sexually transmitted infections.

**Material and methods:**

The data dictionaries were first structured to facilitate mapping to FHIR and semantic terminologies, including ICD-10, SNOMED-CT, LOINC, and RxNorm. FHIR resources and codes were assigned to data dictionary terms. The data dictionary and mappings were used as inputs for a newly developed tool to generate FHIR implementation guides.

**Results:**

Implementation guides for core data requirements for family planning and sexually transmitted infections were created. These implementation guides display data dictionary content as FHIR resources and semantic terminology codes. Challenges included the use of a two-dimensional spreadsheet to facilitate mapping, the need to create FHIR profiles and resource extensions, and applying FHIR to a data dictionary that was created with a user interface in mind.

**Conclusions:**

Authoring FHIR implementation guides is a complex and evolving practice, and there are limited examples for this groundbreaking work. Moving toward machine-readable guidelines by mapping to FHIR and semantic terminologies requires a thorough understanding of the context and use of terminology, an applied information model, and other strategies for optimizing the creation and long-term management of implementation guides. Next steps for this work include validation and, eventually, real-world application. The process for creating the data dictionary and for generating implementation guides should also be improved to prepare for this expanding work.

Funding.

This work was supported by the World Health Organization, which also worked as a collaborative partner throughout the study.

## Highlights

• Clinical Guidelines can be translated into systems in non-standard ways.
• Robust tooling and processes for creating computable guideline are needed.
• FHIR and semantic terminologies facilitate guideline implementation in practice.
• Machine-readable guideline authoring requires context and domain expertise.
• Clinical validation is required before real-world implementation.

## Graphical abstract

**Figure:**
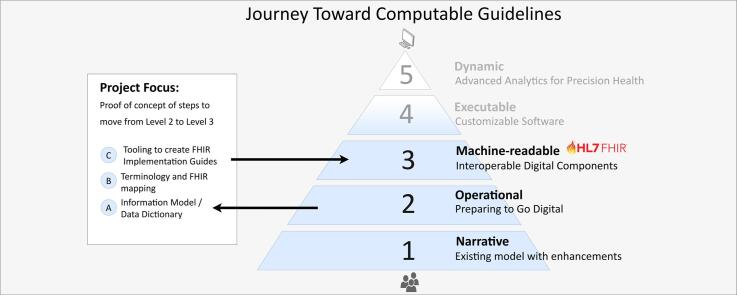

## 1 Introduction

The use of digital technology in health care, such as electronic health records (EHRs), continues to increase as countries work to implement public health interventions, to improve care delivery with decision support, and to ensure accountability at all health system levels. The development of clinical practice guidelines (CPGs) have been a significant enhancement and are used worldwide to inform clinical decision-making through the implementation of evidence-based clinical and public health practices [1], [2]. However, CPGs often contain assumptions, knowledge gaps, and ambiguities that make translation into an electronic computable format difficult, resulting in varied and subjective interpretations of the guidelines, and incomplete or inaccurate implementations and operationalization [3], [4], [5], [6], [7], [8], [9]. These difficulties can lead to divergence in electronic CPG implementations, reducing the usefulness of collected data outside of that implementation setting [1], [2], [3], [4], [10]. Solutions to this challenge have been explored, such as implementing a guidelines-based decision support system [3], [11] or the Arden Syntax [12], but these solutions lack a standards-based focus that can be implemented globally because they do not incorporate consistent data structures and coding schemes and thus require customization [12].

To resolve these challenges, the World Health Organization (WHO), Centers for Disease Control and Prevention (CDC), and others are moving toward machine-readable guidelines better suited for the digital age [13], [14], [15]. WHO has outlined a vision to move toward a model that supports machine-readable guidelines and enables standards-based data exchange and computerized clinical decision support [16], [17]. As one of the first steps in that journey, WHO created Digital Adaptation Kits (DAKs) to define system requirements, including minimum datasets and business processes, for specific areas, such as antenatal care [5], [18]. The creation of a data dictionary based on the DAK helps align implementers with the minimum data needed to support client care delivery and facilitate decision support and indicator calculation. A completed DAK contains the following components:

• Linked health interventions and recommendations
• Personas
• User scenarios
• Business processes and workflows
• Core data elements
• Decision support logic
• Programme indicators
• Functional and non-functional requirements for an implementing system

WHO’s DAKs are designed to facilitate movement from narrative clinical guidelines, L1, to a semi-structured data dictionary in L2 [19], as shown in Table 1. DAKs specify the minimum data set by enumerating the health concepts used in the guideline to specify a data dictionary. While this is one essential step for consistent, standardized computability, additional work is required to move to L3, the machine-readable, software neutral level. It is necessary to enable decision support by structuring WHO’s narrative guidelines with a computer-readable standard (i.e. FHIR) and semantic terminology codes for the data dictionary provided in the DAK. This work did not directly involve interpreting or implementing clinical decision support logic, although it lays the groundwork to enable the use of such logic in low- and middle-income countries (LMICs) and contributed to the finalization of the DAKs. Success in this area involves creating guidelines that are computable upon release from the guidelines authors, i.e. WHO, rather than requiring implementers to interpret the guidelines to make them computable in their own systems.

**Table 1:** Knowledge Layers of Computable Guidelines.

**Knowledge Layer**	**Products**	**Description**
L1: Narrative	Enhanced guidelines	Guidelines and data recommendations
L2: Operational	Digital adaptation kits	Semi-structured documentation of operational and functional requirements
L3: Machine readable	Machine-readable recommendations	Structured, software-neutral, specifications, code, terminology and interoperability standards
L4: Executable	Reference software	Software that is able to execute static algorithms and interoperable digital components and deliver operational and functional requirements
L5: Dynamic	Precision health models	Executable dynamic algorithms that are trained and optimised with advanced analytics to achieve prioritised outcomes

(Adapted from Mehl et al. [16]).

Moving products created in L2 to a structured and software agnostic L3 requires utilizing syntactic standards such as HL7 Fast Healthcare Interoperability Resources (FHIR) and standard semantic terminologies to more precisely define the data required to support the guidelines and tooling to support the creation of the FHIR Implementation Guides (IGs) [6], [20]. FHIR is a platform specification for describing interoperable healthcare data exchange. In particular, it provides the ability to define profiles that provide structure to ensure semantics of the data being exchanged are understood by all parties and support integration and translation of data from different platforms [21]. FHIR IGs provide a publishing mechanism for these profiles. FHIR works closely with semantic terminologies by providing definitions for data structure and leveraging codes and definitions provided by terminologies. Semantic terminologies enable the codified designation of a clinical concept so that the meaning of a particular medical term remains consistent across different digital systems. These terminologies support transfer of meaning when sharing data between disparate computer systems. Examples of these terminologies include Logical Observation Identifiers, Names, and Codes (LOINC) for laboratory values and questionnaires, Systematized Nomenclature of Medicine Clinical Terms (SNOMED-CT), International Classification of Disease (ICD-10), and RxNorm [22], [23], [24], [25]. The terminology and FHIR Resources together form a basis for communicating the structure and meaning of the clinical data. This sets the stage for clinical decision support, which can leverage FHIR’s data structures and semantic terminology standards to ensure decision support logic is implementable.

The goal of this work was to demonstrate feasibility in creating L3 machine-readable standards and to illustrate the feasibility of automatically generating FHIR IGs using L2 artifacts. To establish the processes and resourcing required to successfully create FHIR IGs, the team created a mapping process, set up a collaborative mapping environment, and mapped family planning (FP) and sexually transmitted infections (STI) terms to FHIR and semantic terminologies. To support an automated process for creating the FHIR IGs, the team developed an initial version of tooling to accept mapped terms as inputs and produced FHIR profiles and IGs as outputs.

## 2 Material and methods

### 2.1 DAK dictionary preparation

The DAK data dictionaries were received from WHO as spreadsheets that contained core data elements for FP and STI recommendations. However, these data elements were not yet mapped to semantic terminology or a data standard like FHIR as they were still in an early stage. The modeling of these data dictionaries was based upon a user interface, listing questions with expected data formats and selectable answers where applicable. These data dictionary files were structured as human-readable multi-sheet workbooks that were designed to support practices that are based upon the clinical care continuum. The original structure had multiple sheets with terms potentially repeated based upon when the data would be collected and used in the clinical care workflow. An example of the FP data dictionary can be found on the WHO website, linked in the Appendix.

To ensure consistent mapping to FHIR and semantic terminology across the care continuum, the DAK data dictionary files were consolidated into a single flat “master” data dictionary spreadsheet in Google Sheets. Additional columns for use in mapping and coding were added to the data dictionary. These included columns for calculated fields to group terms, highlight duplicates, enable monitoring of mapping progress, and equip team members with fields for notes and mapping validation. Additional data dictionary attributes necessary for FHIR resource and semantic terminology mapping of terms were added to enable and support use of an automated IG-generation tool, as shown in Table 2.

**Table 2:** Dictionary Attributes Required for FHIR and Semantic Terminology Mapping.

***FHIR Resource Mapping***		
**Attribute**	**Meaning**	**Example**
FHIR Resource	Target FHIR resource type and path(s) to the elements of that resource to which a data element is mapped, also noting resource type and FHIR version where applicable	AllergyIntolerance.code
Additional FHIR Attributes	Attributes to be included in the FHIR profile generated	AllergyIntolerance.category = medication
Target Profile ID	Profile to which a data element belongs in the generated implementation guide, either an existing or a newly created profile	WHO-Core AllergyIntolerance (Drug Allergies)
***FHIR Value Set Mapping***		
Value Set Binding	Existing or custom value set to be used for binding, also noting the strength of the binding if the default “Required” binding strength is not appropriate.	http://hl7.org/fhir/ValueSet/communication-not-done-reason
***Semantic Terminology Mapping***		
FHIR Code System	Code system to which a term is assigned, which is needed only if a “HL7 FHIR R4 Code” is specified.	http://hl7.org/fhir/administrative-gender
FHIR Code	A code specified by FHIR, which resides in a particular code system.	female
Semantic Terminology Code	Code(s) specified in one or more selected terminologies.	ICD-10: N91.2 (maps to Amenorrhea) SNOMED: 14,302,001 (maps to Amenorrhea) LOINC: 11996–6 (maps to Number of Pregnancies) RxNorm: 18,631 (maps to azithromycin)

FHIR resource and profile organization examples were identified from FHIR’s Implementation Guide Registry to align this work with existing implementation guide structure [26]. This work followed the conventions of the International Patient Summary (IPS) [27].

### 2.2 Mapping to FHIR and semantic terminology

**Fig. 1:**
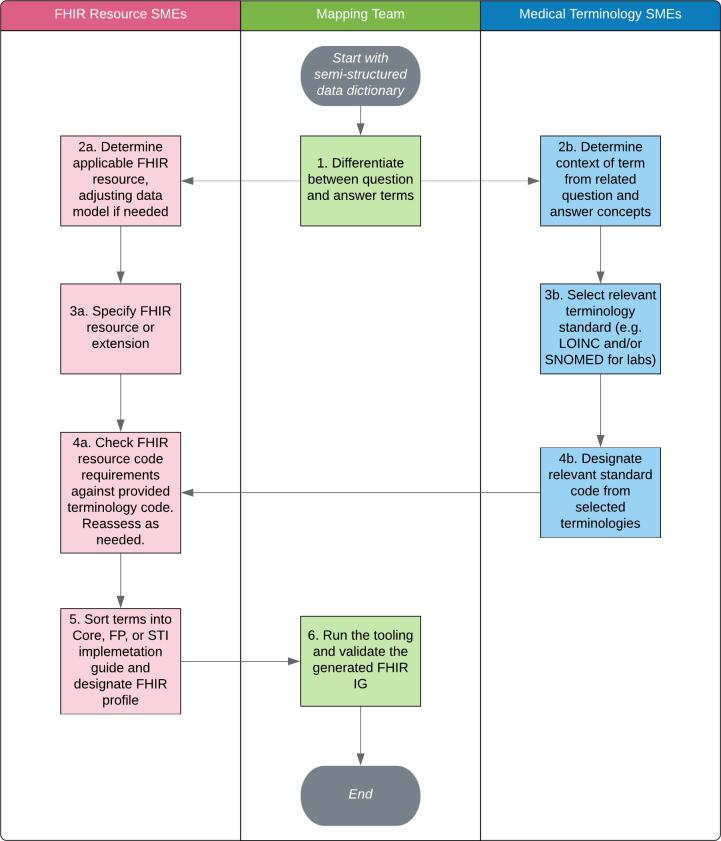
FHIR and Terminology Mapping Process. SME: Subject Matter Expert

The following methods were used to map the DAK data dictionary concepts to the appropriate FHIR Resource type and standard terminologies like LOINC, SNOMED-CT, ICD-10, and RxNorm to represent the health concept, allowing FHIR Profiles and IGs to be generated. A mapping team was assembled with skills in clinical care provision, data modeling, semantic terminology, and FHIR modeling. This team proposed a mapping process to navigate decision points during mapping and assign FHIR and terminology mappings where applicable. This process was tested and iterated multiple times using subsets of terms, making improvements in efficiency and driving toward effective and valid mappings. This process is illustrated in Fig. 1, displaying how the team started with a user-interface-oriented data dictionary without mappings to semantic terminology codes or to FHIR resources. Semantic terminology codes were required to adequately create draft implementation guides, although the project primarily focuses on FHIR implementation guide authoring.

The Unified Medical Language System (UMLS) was used to search for and identify terminology codes since it facilitates terminology mapping across multiple standards [28]. For this work, LOINC, SNOMED-CT, ICD-10, and RxNorm were the chosen semantic standards for mapping unless FHIR could already accommodate mapping with its own code(s).

### 2.3 Using tooling to produce the FHIR IG

At the time of this project, mature tools were not publicly available for generating FHIR implementation guides (IGs), prompting the need for custom development of tooling that could accept a DAK data dictionary with mappings and create files necessary for a FHIR IG. To support subject matter experts' abilities to generate FHIR profiles and the FHIR IG, tooling was developed that used the data dictionary with the added FHIR resource and terminology mappings as inputs to automatically generate the required FHIR IG artifacts. These FHIR IG artifacts as referenced in the Appendix, included the FHIR differential table listing the resources and attributes, profiles and extensions, value sets, and definitions suitable for inclusion in a publishable FHIR implementation guide.

The tooling was created through an iterative process. The first step was to create tooling that produced FHIR Profiles from the “master” data dictionary containing the FHIR resource and standard terminology mappings. Next, the development team worked with the mapping team in an iterative process to establish the FHIR differential table listing the resources and attributes needed to support creation of FHIR profiles. This tooling allows for the FHIR IG to be generated and re-generated as items are added and maintained in the DAK.

### 2.4 Validation of the mappings and FHIR IG

An iterative process was conducted where errors and gaps in the spreadsheet and tooling were regularly discussed and addressed as the team worked to establish tooling requirements and map terminology and FHIR resources. As the team worked through establishing the mapping, the row-based nature of the mapping spreadsheet required duplication of many mapping terms. Each concept in the data dictionary was checked to be present and displayed as intended in the IGs. Discrepancies and errors were noted, addressed, and rechecked.

## 3 Results

For both the FP and STI programs, the project team was able to use a data dictionary with added FHIR resources and semantic terminology mappings to successfully generate a FHIR implementation guide, which contained profiles that each consisted of a differential table from base FHIR. Each IG lists the resources and attributes for a minimum data set, profiles and extensions, value sets and definitions suitable for inclusion in a publishable FHIR implementation guide. The mapping was done through the use of FHIR and semantic terminology mapping processes above to designate the FHIR resource and corresponding data element types. The FHIR resources applied to the project are outlined below [29]. The FHIR resources below were adequate to cover all elements within the data dictionary. As such, these resources could be adapted for this project and no new FHIR profiles needed to be created. Due to the nature of the minimum data set, most of the elements in the minimum data set were mapped to the FHIR Patient, Condition, and Observation resources. The learning from this collaborative effort sets the stage for alignment on the structure of the data elements in the DAK data dictionary and informs the data, processes, and tools required for creation of future FHIR IGs and DAKs.

The following FHIR 4.0.1 Resources [29] were used in this work:

• AllergyIntolerance
• Appointment
• CarePlan
• Communication
• Condition
• Consent
• Coverage
• DeviceUseStatement
• Encounter
• HealthcareService
• Medication
• MedicationAdministration
• MedicationStatement
• Observation
• Patient
• Practitioner
• Procedure
• ServiceRequest
• Specimen

Machine readable guidelines were established through the creation of FHIR IGs that utilized terminology to support semantic meaning and syntactic guidance for the data dictionary elements. The semantic meaning for data dictionary terms supporting observations, medication, procedures, and conditions were specified using applicable medical terminologies that included ICD-10 [24], SNOMED-CT [23], LOINC [22] and RxNorm [25]. A full list of FHIR profiles defined in this project, along with examples of profiles and value sets created, can be found in Table 3. The implementation guides produced by this work (seen in the Appendix) were not published using FHIR’s formal publication process due to the need for further validation of mappings and implementation guides by WHO.

**Table 3:** FHIR Profiles in Core, FP, and STI Implementation Guides

**Core Implementation Guide**	
WHO-Core AllergyIntolerance	WHO-Core Observation (HIV Stage)
WHO-Core AllergyIntolerance (Drug Allergies)	WHO-Core Observation (HIV Status)
WHO-Core Appointment (Return Visit)	WHO-Core Observation (HIV Test)
WHO-Core Appointment (Visit)	WHO-Core Observation (Last Normal Menses)
WHO-Core CarePlan (Follow-up)	WHO-Core Observation (Miscarriage or Abortion)
WHO-Core CarePlan (Intake Contraceptive Method)	WHO-Core Observation (Never used contraception)
WHO-Core CarePlan (Prior Contraceptive Methods)	WHO-Core Observation (Number of births)
WHO-Core Coverage (Insurance)	WHO-Core Observation (Number of Pregnancies)
WHO-Core DeviceUseStatement (Intake Contraceptive Method)	WHO-Core Observation (Postpartum)
WHO-Core DeviceUseStatement (Prior Contraceptive Methods)	WHO-Core Observation (Pregnancy Status)
WHO-Core MedicationStatement (Current Contraceptive Methods)	WHO-Core Observation (PrEP Status)
WHO-Core MedicationStatement (Current Medication)	WHO-Core Observation (Reason for no contraceptive method at intake)
WHO-Core MedicationStatement (Prior Contraceptive Methods)	WHO-Core Observation (Recent Sexual History)
WHO-Core Observation (BMI)	WHO-Core Observation (Sexual History)
WHO-Core Observation (Body Height)	WHO-Core Observation (Sexually Active)
WHO-Core Observation (Body Weight)	WHO-Core Observation (STI Risk Factors)
WHO-Core Observation (Breastfeeding)	WHO-Core Observation (Temperature)
WHO-Core Observation (Clinical Observations)	WHO-Core Observation (Time Postpartum)
WHO-Core Observation (Date of Delivery)	WHO-Core Patient
WHO-Core Observation (Days since Unprotected Sex)	WHO-Core Practitioner
WHO-Core Observation (Gender-based Violence Risk Factors)	WHO-Core Procedure (Intake Contraceptive Method)
WHO-Core Observation (Gender-based Violence Victim)	WHO-Core Procedure (Prior Contraceptive Methods)
WHO-Core Observation (HIV Care Enrollment)	WHO-Core ServiceRequest (Referral)
**Family Planning Implementation Guide**	**Sexually Transmitted Infection Implementation Guide**
WHO-FP CarePlan (Backup Contraceptive Method)	WHO-STI CarePlan (Recommendation)
WHO-FP CarePlan (Exit Contraceptive Method)	WHO-STI Communication (Partner)
WHO-FP CarePlan (Recommendation)	WHO-STI Condition (Diagnosis)
WHO-FP CarePlan (Requested Contraceptive Method)	WHO-STI Condition (HIV)
WHO-FP Condition (Medical Eligibility)	WHO-STI Condition (STI History)
WHO-FP Consent	WHO-STI Medication
WHO-FP DeviceUseStatement (Exit Contraceptive Method)	WHO-STI MedicationAdministration
WHO-FP DeviceUseStatement (Requested Contraceptive Method)	WHO-STI MedicationStatement (Current Medication)
WHO-FP Encounter	WHO-STI MedicationStatement (Medication History)
WHO-FP Encounter (Contraception Issues and Concerns)	WHO-STI Observation (Current STI Treatment)
WHO-FP HealthcareService	WHO-STI Observation (Disease Exposure)
WHO-FP MedicationStatement (Current Medication)	WHO-STI Observation (External Genital Examination)
WHO-FP MedicationStatement (Exit Contraceptive Method)	WHO-STI Observation (Increased STI Risk)
WHO-FP MedicationStatement (Requested Contraceptive Method)	WHO-STI Observation (LGV Follow-up)
WHO-FP Observation (Breastfeeding Status)	WHO-STI Observation (Partner HIV Status)
WHO-FP Observation (Days since Last Normal Menses)	WHO-STI Observation (Partner Symptoms)
WHO-FP Observation (Intercourse since Last Normal Menses)	WHO-STI Observation (Past STI Treatment)
WHO-FP Observation (Medical eligibility number)	WHO-STI Observation (Recent Abortion or Miscarriage)
WHO-FP Observation (Medical eligibility text)	WHO-STI Observation (Recent Syphilis Treatment)
WHO-FP Observation (Medical Eligibility)	WHO-STI Observation (Reported Symptoms)
WHO-FP Observation (Missed or Late Menses)	WHO-STI Observation (Risk Assessment)
WHO-FP Observation (Pregnancy Intention)	WHO-STI Observation (Signs of Fever)
WHO-FP Observation (Reason for no contraceptive method at exit)	WHO-STI Observation (Signs of Shock)
WHO-FP Observation (Reason for Stopping Contraception)	WHO-STI Observation (Speculum Examination)
WHO-FP Observation (Smoking Status)	WHO-STI Observation (STI Screening)
WHO-FP Observation (Test Results)	WHO-STI Observation (Syphilis Stage)
WHO-FP Procedure (Exit Contraceptive Method)	WHO-STI Observation (Trauma History)
WHO-FP Procedure (Requested Contraceptive Method)	WHO-STI Patient
WHO-FP Procedure (Service Provided)	WHO-STI Procedure (Service Provided)
	WHO-STI Specimen

### 3.1 Challenges

As the team worked through establishing the mappings, the row-based, two dimensional nature of the mapping spreadsheet required duplication of many of the mapping terms. The team worked through approaches for reducing that duplication by allowing, for example, answer value sets to be specified one time, and then referenced by any appropriate data elements. The challenges of supporting the complex content in a two dimensional structured spreadsheet also impacted the tooling. For example, when multiple terminology codes were applicable to a term, the tooling was forced to parse through text, whereas an improved structure would have more effectively facilitated the use of multiple terminology codes.

Another challenge was determining a process for evaluating if an element was adequately covered by an existing FHIR profile, versus the need to define a new profile. We established a shared “core” implementation guide to support defining common profiles between FP and STI, and enabled the tooling to organize profiles and value sets into their respective implementation guides.

**Fig. 2:**
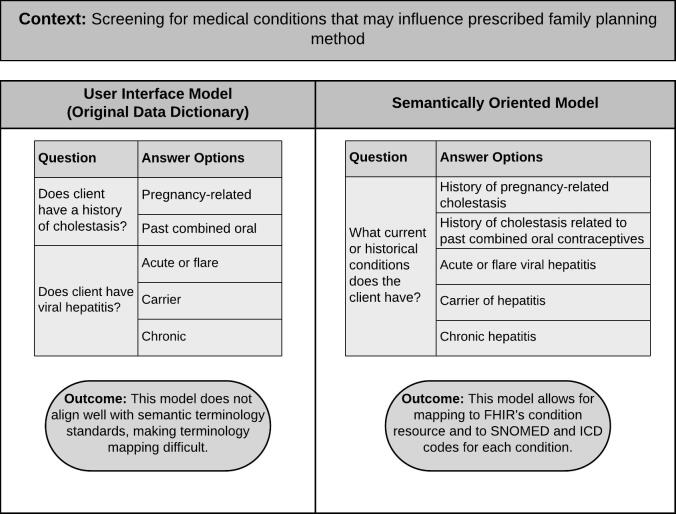
Example of Information Modeling.

Another major challenge was the need to apply a formal information model (i.e., FHIR) to an already established data dictionary, which was primarily built with user interaction in mind. This required the mapping team to alter the original data dictionary’s implicit data modeling to fit into a more FHIR-ready model, as shown in step 2a of Fig. 1. Fig. 2 below displays an example where the implicit, user-interface-oriented model required altering. Many of the data remodeling challenges required working directly with WHO’s clinical guidelines experts to understand the context of the term and whether or not each proposed remodel was appropriate for this context. While the DAK data dictionary was built for the end user, the FHIR model is designed to enable machine-readable guidelines to be implemented by system engineers, developers, etc. in point-of-care systems. This model facilitates the mapping of data dictionary elements to FHIR resources and to semantic terminology codes, such as an ICD code for a disease. The value of the User Interface Model is that it is easy for users to interact with when using a form or an EHR, while the Semantically Oriented Model aligns with data and terminology standards. Creating the Semantically Oriented Model from the User Interface Model required additional information and context to help move the DAK toward L3; however, this model can be easily reverted back to the User Interface Model when displaying for a user.

## 4 Discussion

### 4.1 Key learning

**Fig. 3:**
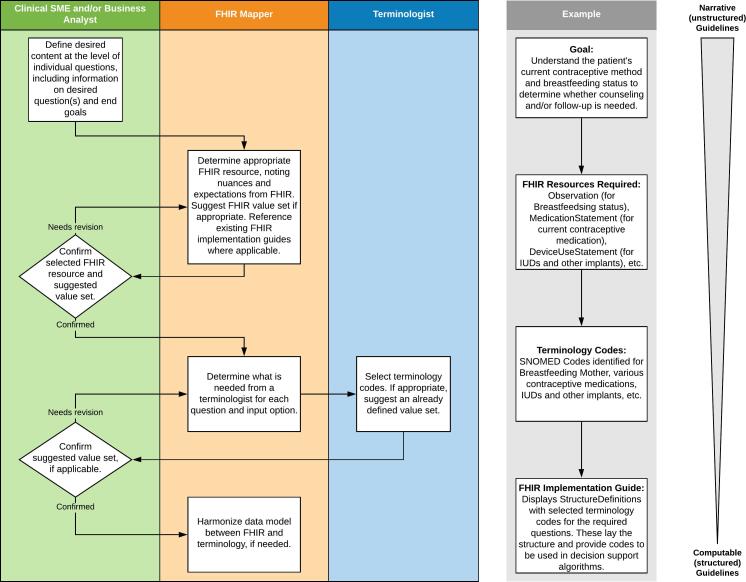
Recommended Process for Data Dictionary Creation and Mapping to FHIR and Terminology.

The WHO FP and STI programs are among the first projects to build upon WHO’s narrative guidance documents toward establishing machine-readable guidelines, and this project piloted the creation of machine-readable L3 artifacts from L2 DAKs. While the work was successful in proving that a FHIR Implementation Guide can be created from a form-based, user interface-driven data dictionary, the process described above was developed based upon the work which preceded it, and the assumptions inherent in that work. In particular, this work was based upon the unpublished version of the FP and STI DAKs and informed the need to ensure that terminology was included in the DAK creation process. While most of these process steps are inherently necessary steps in a full implementation guide development process, it is recommended to reconsider the overall process for creating L2 and L3 artifacts and when the various actors engage with each other. Ideally, the process described above changes for subsequent DAK work in other domain areas. Fig. 3 gives a proposed process to enhance DAK data dictionary creation.

Because of the FHIR community’s increasing need for FHIR IG creation and maintenance, the available tools for this have recently matured and replaced the need for the custom tooling used in this project [30]. In particular, FHIR Shorthand (FSH) is a language specifically for the creation of these implementation guides, with guidance and training available [31], [32].

The following are some of the key learning points and discussion points that should be considered in future projects that include semantic terminology mapping to create machine-readable (L3) artifacts like FHIR IGs:

1. **The best quality mapping requires understanding the concept’s use from data entry through decision support.** To effectively codify concepts using standard medical semantic terminologies and appropriate FHIR Resources, the mapping team needs to understand the concepts from data entry of the concept through the use of the concept for decision support. This perspective of data capture through data use is necessary to understand the clinical intent of the data being captured and assist in clearly naming the concept and mapping it appropriately. The passing of information from one person or system to another leads to a loss of information, so it is important to work with the creators of the data dictionary (i.e. WHO guideline SMEs) to prevent loss of fidelity of information. This applies both in the exchange of information and in the creation of L2 and L3 artifacts.
2. **It is important to have an information model or defined pattern for mapping to FHIR.** Many of the data dictionary terms were stated in the physical way that they were represented on a physical form or screen used during care. For example, “hypertension” with a yes/no answer set could be modeled as a FHIR Observation resource that is linked to one semantic terminology code with “yes” or “no” as the answer set. However, one could also model hypertension in a FHIR Condition resource. This allows for capturing any number of medical terminology codes that relate to hypertension or any other potential patient current conditions. Both of these modelings allow for the capture of hypertension, but the first is focused on identifying a “yes” or “no” for a specific code and the second allows for creating a value set for multiple hypertension codes and other codes that can be used to identify a patient’s current conditions. Alignment on the underlying information model should be a part of guidance and, at a minimum, should be used to inform the creation of the DAK data dictionaries.
3. **It is important to have a semantic terminology mapping strategy.** A considerable amount of time was dedicated to mapping the data dictionary terms to semantic terminologies, and there are some key decisions that can help guide these choices. Determining which semantic terminologies to include for which types of dictionary terms is important. For example, identifying which semantic terminologies will be used to identify medical conditions and which will be used for medications will set the scope for the work. Secondly, it is important to have a strategy that addresses how general or specific terms in a value set should be. The team will need to determine if they are identifying a comprehensive value set for each term in the dictionary, for example, exhaustively including all diabetes terms or if a higher level or all-inclusive term is sufficient to support the desired use cases.
4. **There needs to be a long-term management strategy to support an Implementation Guide.** The process for this work started with a WHO Digital Adaption Kit that included a data dictionary. The data dictionary was then organized and augmented to enable the creation of the FHIR Implementation Guide. This was done using a spreadsheet. The team documented the use of many of the columns, but there is still information that must be elucidated to allow others to maintain and support the work moving forward. The use of a tool for managing the data dictionary is recommended. A terminology or data dictionary management tool that supports more robust data structures than a spreadsheet is recommended, such as a terminology service. In addition, there is a need to be able to manage versioning and to manage updates to the narrative guidelines moving forward.
5. **Clinical semantic terminology mapping may be more accurate and usable if done as a part of the guidance creation or Digital Adaption Kit process.** CPGs require as much accuracy as possible to ensure clinical quality. Mapping existing forms and instruments to standards inevitably uncovers dissonance or a lack of clarity between concepts used in the data entry forms used in daily health care practices and existing international standards**.** This project supported coding after the data dictionary had been created. It is recommended that an approach that supports terminology mapping as a part of guidance update or the Digital Adaptation Kit creation process would result in more accurately representing the clinical data needed to support both the data collection and analytics.
6. **Not all FHIR Implementation Guides have the same purpose.** FHIR Implementation Guides created to date have been created for specific purposes like data exchange, querying, or defining minimum data sets. Because of the diverse uses, care must be taken when looking at patterns for creating an implementation guide. It is also important to identify the purpose of the guide that is being developed.

## 5 Conclusions

The transition from narrative clinical guidelines to machine-readable implementation guides is complex and still evolving. At the time of this project, few and limited examples were available to inform approaches for the transition of human-readable guidelines into machine-readable guidelines, especially when modeling after business processes and user interface. Explorations in this space, including this project, have already yielded valuable insights into how the generation of FHIR IGs and computer readable technical guidance creation can evolve and improve, especially as it relates to data exchange and semantic requirements through the various levels and teams involved in the creation of guidelines and related content.

Next steps for this work include validation of mappings and implementation guides, which will allow for real world application. This validation may include testing within medical record systems and use in clinical decision support algorithms. It is also recommended that the proposed process for implementation guide generation is iterated upon for the sake of efficiency once this work is expanded. In particular, incorporating technologies, tools, and practices into this process that support the creation, versioning, management, and publication of these data elements and value sets will enhance sustainability of the work as it becomes used broadly by global implementers. DAKs in other areas of care will also be developed using a more optimized process, based on the lessons learned from this work. Legacy point-of-care applications may require mapping to the computer-readable guidance in order to achieve interoperability.

## CRediT authorship contribution statement

**Jennifer Shivers:** Methodology, Project administration, Writing – original draft. **Joseph Amlung:** Data curation, Methodology, Writing – original draft. **Natschja Ratanaprayul:** Conceptualization, Funding acquisition, Writing – review & editing. **Bryn Rhodes:** Software, Writing – review & editing. **Paul Biondich:** Supervision, Validation.

## Declaration of Competing Interest

The authors declare that they have no known competing financial interests or personal relationships that could have appeared to influence the work reported in this paper.

## Figures and Tables

**Figure:**
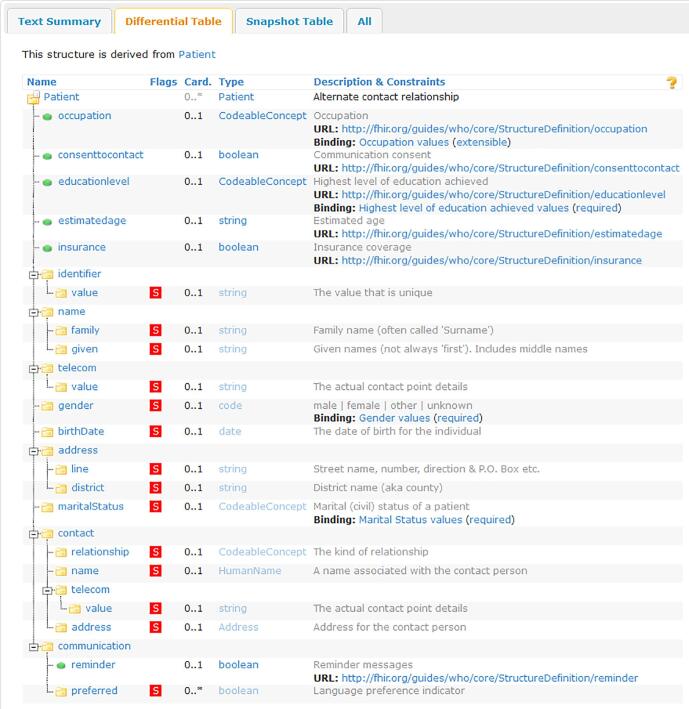
Example Profile: WHO-Core Patient

**Figure:**
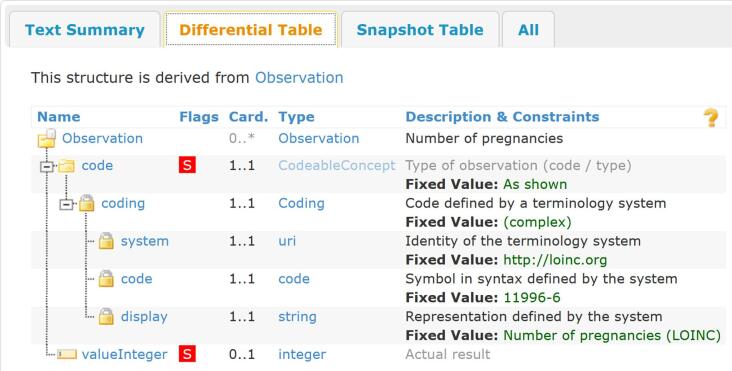
Example Profile: WHO-Core Observation (Number of Pregnancies)

**Figure:**
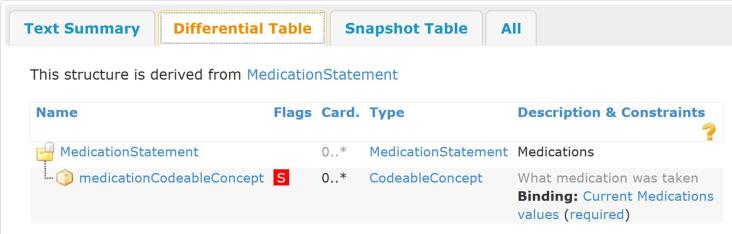
Example Profile: WHO-FP MedicationStatement (Current Medication)

**Figure:**
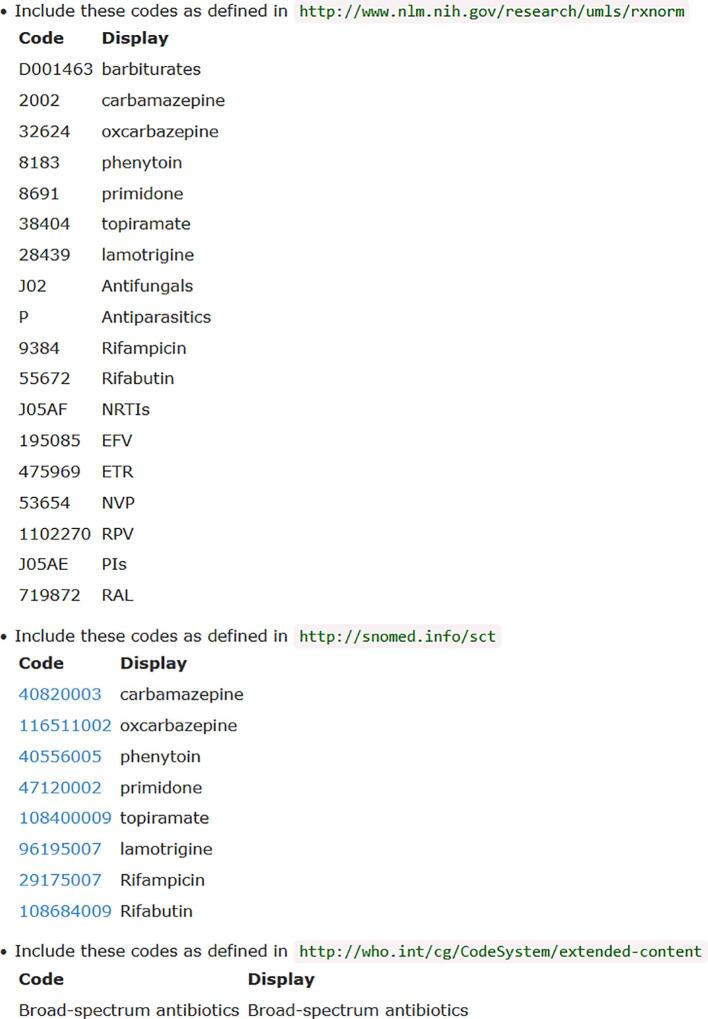
Example Value Set: WHO-FP MedicationStatement (Current Medication)

## Appendix Examples

### WHO Family Planning Digital Adaptation Kit (including Data Dictionary)

The WHO Digital Adaptation Kit for Family Planning was released in the time between the completion of this work and the writing of this publication. Some minor updates have been made to the data dictionary itself since the FHIR Implementation Guides were created, based on lessons learned from this work. However, it still can serve as an example of the starting point from which this work was based.

FP DAK: https://www.who.int/publications/i/item/9789240029743

### WHO Core, Family Planning, and STI Implementation Guides

Note that these links are to the current iteration of the implementation guides, which require validation before real-world implementation. These were not published using FHIR’s formal publication process. These links may expire as the implementation guides continue to iterate.

Core IG: http://build.fhir.org/ig/WorldHealthOrganization/smart-core/.

FP IG: http://build.fhir.org/ig/WorldHealthOrganization/smart-fp/.

STI IG: http://build.fhir.org/ig/WorldHealthOrganization/smart-sti/.
